# A LONGITUDINAL EXAMINATION OF THE EFFECT OF RESILIENCE AGAINST ANXIETY DURING COVID-19

**DOI:** 10.1093/geroni/igad104.2141

**Published:** 2023-12-21

**Authors:** Dawn Carr, Natalie Sachs-Ericsson, Julia Sheffler, Melissa Meynadasy, Brad Schmidt, Greg Hajcak

**Affiliations:** Florida State University, Tallahassee, Florida, United States; Florida State University, Tallahassee, Florida, United States; Florida State University College of Medicine, Tallahassee, Florida, United States; Florida State University, Tallahassee, Florida, United States; Florida State University, Tallahassee, Florida, United States; Florida State University, Tallahassee, Florida, United States

## Abstract

This longitudinal study of community dwelling older adults (N = 453) examined consequences of COVID-related worries on changes in anxiety symptoms before relative to during the pandemic. We further evaluated if pre-COVID psychological resilience (PR) buffered the impact of COVID-related worry. Pre-COVID data were collected in September 2018. COVID-related worry and COVID anxiety symptoms were collected in October 2020 (Wave 2). Controlling for pre-COVID anxiety symptoms, we examined if COVID-related worries (e.g., I’m worried that I might die from COVID-19) were associated with increased anxiety symptoms, and whether pre-COVID PR moderated the association between COVID-related worries and prospective increases in anxiety symptoms. COVID-related worries were associated with increased anxiety symptoms (β = 0.005, p < .01), whereas pre-COVID PR was associated with a decrease in anxiety symptoms (β = -0.029, p < .05). PR moderated the association; COVID-related worries were associated with greater increases in anxiety symptoms among those with low pre-COVID PR (Model η2 = 0.35). Thus, the extent to which COVID-related worries influenced psychological health was dependent on pre-COVID levels of PR. We conclude the combined vulnerabilities of low pre-COVID PR and high COVID-related worries significantly increased the psychological consequences of COVID-19 for our sample of older adults.

